# Immunosenescence profile and expression of the aging biomarker (*p16*^*INK4a*^*)* in testicular cancer survivors treated with chemotherapy

**DOI:** 10.1186/s12885-020-07383-2

**Published:** 2020-09-14

**Authors:** Maria T. Bourlon, Hugo E. Velazquez, Juan Hinojosa, Luis Orozco, Ricardo Rios-Corzo, Guadalupe Lima, Luis Llorente, Diego F. Hernandez-Ramirez, Francisco J. Valentin-Cortez, Irene Medina-Rangel, Yemil Atisha-Fregoso

**Affiliations:** 1 Department of Hematology and Oncology, Instituto Nacional de Ciencias Médicas y Nutrición Salvador Zubirán, Mexico City, Mexico; 2 Escuela de Medicina, Universidad Panamericana, Mexico City, Mexico; 3 Department of Immunology and Rheumatology, Instituto Nacional de Ciencias Médicas y Nutrición Salvador Zubirán, Mexico City, Mexico; 4 Tecnologico de Monterrey, Escuela de Medicina y Ciencias de la Salud, Monterrey, NL Mexico; 5 Institute of Molecular Medicine, Feinstein Institutes for Medical Research, Manhasset, NY USA

**Keywords:** Testicular cancer survivors, Germ cell tumors, Immunosenescence, Premature aging, p16INK4a, Testicular cancer, Molecular aging

## Abstract

**Background:**

Cytotoxic chemotherapy can cure advanced germ cell tumors. Nevertheless, cancer treatment may induce cellular senescence and accelerate molecular aging. The aging process implies an increase of cells expressing *p16*^*INK4a*^ and changes in lymphocyte subpopulations. Our aim was to study the potential induction of premature immunosenescence in testicular cancer survivors (TCS) exposed to chemotherapy.

**Methods:**

Case-control exploratory study of TCS treated with chemotherapy (≥3 BEP cycles, disease-free ≥3 months) compared with age matched healthy controls. Peripheral blood mononuclear cells were isolated, and lymphocyte subpopulations were analyzed by flow cytometry. CDKN2A*/p16*^*INK4a*^ expression in T cells was measured using qPCR. The percentage of lymphocyte subpopulations and the CDKN2A/*p16*^*INK4a*^ expression in TCS were compared with the control group using the Wilcoxon signed-rank test.

**Results:**

We included 16 cases and 16 controls. The median age was 27 years (minimum 24, maximum 54) and the median time on surveillance was 26.5 months (minimum 3, maximum192). TCS had a lower percentage of total T cells and CD4+ T cells in total lymphocytes. Among the CD4+ T lymphocytes, TCS had less naïve CD4+ and increased memory CD4+ cells. Within the CD8+ T lymphocytes, TCS exhibited a decrease in the percentage of naïve cells and an increase in CD8 + CD45RA + CD57+ cells. TCS also exhibited decreased memory CD19+ B cells compared to the controls. The relative expression of CDKN2A*/p16*^*INK4a*^ in T cells was increased in TCS (mean 1.54; 95% CI of the mean: 1.074–2.005; *p* = 0.048).

**Conclusion:**

In this exploratory study, TCS showed increased expression of CDKN2A*/p16*^*INK4a*^ and a lymphocyte phenotype that has been associated with immunosenescence. Further studies are warranted to define the clinical implications of these alterations in TCS.

## Background

Germ Cell Testicular Cancer (TC) mainly affects young males between 15 and 35 years old [1]. It is estimated that there were 71,105 new cases and 9507 deaths worldwide in 2018 [2]. It is the most curable solid neoplasm, and even if it presents in advanced stages, it can still be cured with current chemotherapy regimens [3]. According to the International Germ Cell Consensus Classification (IGCCC), more than 90% of patients with good risk disease will be cured, the intermediate-risk group has a 5-year survival rate of approximately 80%, and patients with the poor-risk disease have a 5-year survival rate of nearly 50% [4].

However, testicular cancer survivors (TCS) struggle with the long-term toxicity of oncologic treatment. Side effects include increased incidence of secondary malignancies, hypogonadism, pulmonary toxicity, nephrotoxicity, neurotoxicity, augmented mortality from circulatory diseases, and an increased risk of infections [5, 6]. TCS have a higher risk of dying from non-cancerous causes, including infections, digestive diseases, respiratory and circulatory diseases, than the general population [6]. The aging process within the immune system is called immunosenescence. The clinical manifestations associated with immunosenescence in the elderly include a decreased ability to control infections, poor response to vaccination, and an increment in the risk of developing cancer [7]. Several immunological alterations have been described with aging, including changes in T and B lymphocytes subpopulations and natural killer (NK) cells [7]. Of note, there is a decrease in naïve CD4+/CD8+ ratio and an increase in memory T cells relative to naïve [7]. CD4+ CD28- T cells, a unique type of proinflammatory T cells that lack expression of the costimulatory CD28 receptor, accumulate in healthy individuals older than 65 years old, whereas healthy young people have only a few CD4 + CD28- T cells [8]. The elderly population also experiences an increase in CD57+ terminally differentiated senescent cells, which have a reduced proliferative capacity and altered functional properties [9]. The B lymphocyte compartment also shows a decrease in naïve B cells with a reciprocal increase in memory B cells [10]. Aging is also characterized by a decrease in circulating antibody levels [10]. At the functional level, NK cells exhibit decreased cytotoxicity, and NK T cells reduced migratory capacity [11].

*p16*^*INK4a*^, a cell-cycle regulating protein and an inhibitor of cyclin-dependent kinases 4 and 6, plays an important role in cellular aging and premature senescence and has been recognized as an aging biomarker [12]. *p16*^*INK4a*^ expression in peripheral blood T cells has been described to sharply increase with chronological age and contributes to an age-induced functional decline of certain self-renewing compartments [13].

Cytotoxic chemotherapy can induce cellular senescence and accelerate molecular aging in cancer cells [14]. However, this effect in non-tumoral cells has not been fully addressed. Changes in lymphocyte phenotype and an increased expression of *p16*^*INK4a*^ in CD3+ lymphocytes have been reported in breast cancer survivors after adjuvant chemotherapy [15]. To date, this phenomenon has not been studied in TCS population; a group of patients that warrants special attention given the fact that they develop neoplastic disease and receive oncologic treatments at a very young age, and premature immunosenescence may impose many consequences during their lifespan.

Our aim was to evaluate the impact of chemotherapy on the peripheral lymphocyte population and expression of CDKN2A*/*p16INK4a, in order to assess premature immunosenescence.

## Methods

A case-control exploratory study of testicular cancer survivors (TCS) treated with chemotherapy matched by age and gender with healthy controls. Cases were defined as TCS, 18 years old or older, who were in surveillance and no evidence of disease (NED) ≥ 3 months (negative tumor markers and computed tomography (CT) image with no evidence of disease after the last oncologic treatment), and who had received at least 3 BEP (bleomycin, etoposide, and cisplatin) cycles. Patients treated with high dose chemotherapy and bone marrow transplant were excluded. Controls were healthy males, with no previous history of cancer, matched by age (+/− 12 months) in a 1:1 ratio. Before the inclusion in the study and in order to be considered “healthy”, all participants were evaluated clinically by an Internal Medicine specialist (MTB) and screened for type 2 diabetes mellitus and dyslipidemia.

This study was approved by the Institutional Biomedical Research Board of the Instituto Nacional de Ciencias Médicas y Nutrición Salvador Zubirán (REF. 1785). All subjects were informed about the objectives of the study and gave their written consent to participate.

### Isolation of peripheral blood mononuclear cells (PBMC)

A sample of venous blood (40 mL) was obtained from each subject. PBMC were isolated by gradient centrifugation using Lymphoprep (Axis-Shield PoC AS, Oslo, Norway). Researchers were blinded to the origin of the sample cases vs controls.

### CD3 + -cell purification

CD3-mAb-coated microbeads (Miltenyi Biotec, Bergisch Gladbach, Germany) were used to purify CD3+ cells by positive selection following the manufacturer’s instructions. Purity was assessed by flow cytometry with an anti-human CD3-FITC monoclonal antibody. This procedure normally yielded CD3+ T-cell preparations with purity > 95%.

### RNA extraction and cDNA synthesis

Total RNA from CD3+ lymphocytes was obtained using Trizol (Life Technology, New York, USA) according to the manufacturer’s instructions. cDNA was synthesized from total RNA by using random hexamers as primers and murine leukemia virus reverse transcriptase (RT) following the manufacturer’s protocol (Invitrogen, Carlsbad, CA, USA).

### Expression of CDKN2A*/p16*^*INK4a*^

The expression of CDKN2A*/p16*^*INK4a*^ was measured using the qPCR Taqman assay (TaqMan Universal Master Mix II, with UNG, Applied Biosystems, Foster City, USA) according to the manufacturer’s specifications. TaqMan probes were used for CDKN2A*/p16*^*INK4a*^ (P16-FAM HS_00924091), Applied Biosystems, Foster City, USA) and 18S (18S-VIC HS_99999901) (Applied Biosystem, Foster City, USA). The samples were performed in duplicate in the real-time polymerase chain reaction (RT-PCR) equipment Corbett Research model RG-6000 (Sydney, Australia) using the program Roto-gene 6000 version 1.7. The relative expression in cases vs controls of CDKN2A*/p16*^*INK4a*^ was analyzed using the 2-ΔΔCt method comparing each case with a matched control.

### Immunophenotyping of leukocyte subpopulations

EDTA-treated blood samples were analyzed by 8-color flow-cytometry (Becton Dickinson Canto II Cytometer) using fluorescence-labelled antibodies from Biolegend Inc. (San Diego, USA). Briefly, 250 μL of blood was incubated with fluorochrome-conjugated antibodies for 20 min at room temperature prior to lysis (RBC Lysis Buffer, Biolegend Inc., San Diego, USA) and fixed with 3% formaldehyde/PBS. Leukocytes populations were defined by the following marker combinations: B cells CD3- CD19+, T cells CD3+, CD4 T cells CD3 + CD4+, CD8 T cells CD3 + CD8+, CD4 naïve CD4 + CD45RA + CD197+, CD4 central memory (TCM) CD4 + CD45RO + CD197+, CD4 effector memory (TEM) CD4 + CD45RO + CD197-, CD8 naïve CD3 + CD8 + CD45RA + CD197+, CD8 TCM CD3 + CD8 + CD45RO + CD197+, CD8 TEM CD3 + CD8 + CD45RO + CD2197-, NKT cells CD4 + CD56 + CD16high, NK cells CD4-CD56+, naïve B cells CD19 + CD20 + CD27-, Memory B cells CD19 + CD20 + CD27+, Plasmablasts CD19 + CD20-CD27 + CD38high, CD4 Treg cells CD4 + CD127lowCD25+, and CD8 Treg cells CD8 + CD28-.

OneFlow™Setup Beads (BD Biosciences, San Jose, CA, USA) were used to adjust instrument settings, set fluorescence compensation, and check instrument sensitivity. ‘Fluorescence minus one’ controls were used to determine positive and negative staining boundaries for each fluorochrome. Five hundred thousand events were recorded for each sample and analyzed with the FlowJo® software v.10. (FlowJo, LLC., Ashland, OR). For initial gating, singlets were identified using the FSC-Height (FSC-H) by FSC-Area (FSC-A) scatter plot. Then the lymphocyte population was gated in a plot SSC-A versus FSC-A. From there, subsequent gating was designed to identify major populations.

#### Statistical methods

Cases and controls were compared with the Wilcoxon signed-rank test, a *p-*value ≤0.05 was considered statistically significant. Results are expressed as median and interquartile range (IQR) unless otherwise indicated. For analysis of significance of the relative expression of CDKN2A*/*p16INK4a in cases vs controls, Wilcoxon signed rank test was used. SPSS® version 21.0 and GraphPad prism v.7.05 were used for the analysis.

## Results

### Demographics and clinical characteristics

We included 16 cases and 16 controls. The median age was 27 years old (min-max 24–54), there was no difference in the age of cases and controls at inclusion in the study. Median time on surveillance for testicular cancer survivors (TCS) was 26.5 (min-max 3–192) months, and the most common histology was non-seminoma (62.5%). At diagnosis, all patients were on clinical stage II or III, International Germ Cell Consensus Classification (IGCCC) risk was good, intermediate or poor in 62.5, 31.2, and 6.2%, respectively. As shown in Table 1, 75% (*n* = 12) of patients received 3 or 4 cycles of BEP, 25% (*n* = 4) received additional therapy with VIP or TIP. Only 3 patients (18.7%) received radiation therapy because of residual retroperitoneal disease. Fasting glucose was evaluated, and none of the individuals in both groups had diabetes. Testosterone levels were within the normal levels among TCS. Tobacco exposure was reported in 25% of TCS and 18.7% of controls.

**Table 1:** Clinical characteristics of cases

Clinical characteristics	Cases (***n*** = 16)
**Median age (yr) at inclusion (min-max)**	27 (24–54)
**Histology**	
-Seminoma	6 (37.5%)
-Nonseminoma	10 (62.5%)
**Clinical stage at diagnosis**	
-IIB	4 (25%)
-IIC	3 (18.7%)
-IIIA	1 (6.2%)
-IIIB	5 (31.2%)
-IIIC	3 (18.7%)
**IGCCC**^a^ **Risk Classification**	
-Good	10 (62.5%)
-Intermediate	5 (31.2%)
-Poor	1 (6.2%)
**Chemotherapy regimen**	
-BEP for 3 cycles	6 (37.5%)
-BEP for 4 cycles	6 (37.5%)
-BEP for 3 cycles + TIP for 4 cycles	1 (6.2%)
-BEP for 4 cycles + TIP for 4 cycles	2 (12.5%)
-BEP for 3 cycles + VIP for 2 cycles	1 (6.2%)
**Radiation therapy**	
-Yes	3 (18.7%)
**Smoking**	
-Yes	4 (25%)
**Median testosterone (ng/mL) levels (min-max)**	3.75 (1.96–4.91)
**Median time on surveillance (months) (min-max)**	26.5 (3–192)

^a^*IGCCC* International Germ Cell Consensus Classification

### Lymphocyte subpopulations

TCS had a lower percentage of total T cells CD3+ (62.3% (53.8–68.2) vs 73.3% (65.4–81.5) *p* = 0.017) and CD4+ T cells (34.4% (27.9–41.5) vs 42.8% (34.5–52.8) *p* = 0.024) cells in total lymphocytes compared to controls. TCS also exhibited a higher percentage of natural killer T (NKT) cells (3.2% (1.0–12.0) vs 1.0% (0.7–2.8) *p* = 0.049) compared to healthy individuals. No differences were observed in NK or plasmablasts. Results are summarized in Table 2.

**Table 2:** Analysis of lymphocyte subpopulations by flow cytometry

Lymphocyte subpopulations	Cases	Controls	P
CD3+ T (%)	62.3 (53.8–68.2)	73.3 (65.4–81.5)	0.017
CD4+ T (%)	34.4 (27.9–41.5)	42.8 (34.5–52.8)	0.024
CD8+ T (%)	21.1 (14.5–25.1)	23.1 (15.1–29.1)	0.820
NK (%)	9.0 (3.8–14.1)	6.8 (2.1–9.6)	0.278
NKT (%)	3.2 (1.0–12.0)	1.0 (0.7–2.8)	0.049
CD19+ B cells (%)	13.0 (9.0–16.0)	9.7 (7.2–15.1)	0.532

Cells are reported as % of total lymphocytes. Values in cases and controls are reported as median (IQR)

In the CD4+ T lymphocytes subpopulations (Table 3), TCS had a lower percentage of naïve CD4+ cells (33.1% (15.9–44.3) vs 39.2% (31.4–55.7) *p* = 0.026) and an increased percentage of effector memory CD4+ cells (18.1% (13.5–25.8) vs 9.8% (6.8–11.6) *p* = 0.001). A decreased proportion in CD4 + CD28+ in TCS was observed, but did not reach statistical significance (91.7% (85.4–97.3) vs 98.5% (93.8–99.2) *p* = 0.07). No significant differences were observed in the CD4 + CD57+ cells.

**Table 3:** Analysis of CD4+ T cells, CD8+ T cells and B cells subpopulations by flow cytometry

Lymphocyte subpopulations	Cases	Controls	p
**CD4+ T cells subpopulations**^a^			
Naïve (%)	33.1 (15.9–44.3)	39.2 (31.4–55.7)	0.026
Central Memory (%)	38.7 (24.4–48.6)	31.9 (24.4–40.9)	0.569
Effector Memory (%)	18.1 (13.5–25.8)	9.8 (6.8–11.6)	0.001
CD4+ CD28+ (%)	91.7 (85.4–97.3)	98.5 (92.8–99.2)	0.070
CD4+ CD57+ (%)	65.1 (21.2–80.0)	38.1 (24.0–70.6)	0.215
**CD8+ T cells subpopulations**^b^			
Naïve (%)	17.6 (8.3–27.1)	27.0 (19.8–41.1)	0.039
Central Memory (%)	2.2 (1.4–4.4)	4.1 (2.5–7.2)	0.079
Effector Memory (%)	34.0 (21.1–44.1)	31.8 (21.4–37.8)	0.535
CD8+ 45RA+ (%)	60.9 (40.0–67.6)	63.6 (46.8–73.8)	0.717
CD8+ CD28+ (%)	83.5 (57.2–91.7)	83.7 (57.1–93.2)	0.959
CD8+ CD57 (%)	32.6 (18.2–40.1)	20.2 (14.3–26.5)	0.088
CD8+ CD45RA+ CD28- (%)	16.8 (9.0–40.4)	18.45 (7.5–34.4)	0.605
CD8+ CD45RA+ CD57+ (%)	41.6 (22.2–55.6)	24.7 (10.1–32.2)	0.015
**B cells subpopulations**^c^			
Naïve (%)	75.9 (64.3–87.5)	71.0 (65.3–77.8)	0.587
Memory (%)	18.2 (8.0–23.4)	27.8 (22.3–31.8)	0.017
Plasmablasts (%)	0.5 (0.1–1.1)	0.2 (0.1–0.4)	0.163

^a^ CD4+ subpopulations are reported as % of CD4+ cells. Values in cases and controls are reported as median (IQR)

^b^ CD8+ subpopulations are reported as % of CD8+ cells. Values in cases and controls are reported as median (IQR)

^c^ CD19+ subpopulations are reported as % of CD19+ cells. Values in cases and controls are reported as median (IQR)

In the CD8+ T lymphocytes subpopulations (Table 3), TCS exhibited a decrease in the percentage of naïve cells (17.6% (8.3–27.1) vs 27.0% (19.8–41.1) *p* = 0.039) and an increased percentage of CD8+ CD45RA+ CD57+ cells (41.6% (22.2–55.6) vs 24.7% (10.1–32.2) *p* = 0.015). Both groups were similar in other populations analyzed.

Finally, in the B lymphocytes subpopulations (Table 3), TCS exhibited decreased in memory CD19+ B cells (18.2% (8.0–23.4) vs 27.8% (22.3–31.8) *p* = 0.017) compared to controls. No additional statistically significant differences were observed in naïve cells or plasmablasts.

### CDKN2A*/p16*^*INK4a*^ expression

**Fig. 1:**
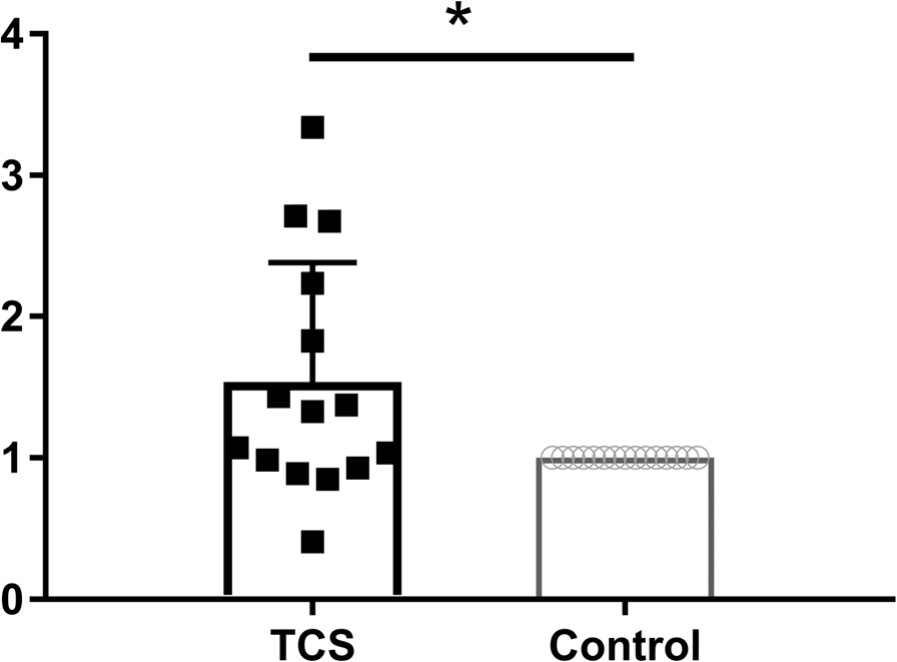
Relative expression of CDKN2A/p16INK4a in testicular cancer survivors. Relative expression of CDKN2A/p16INK4a measured by qPCR in testicular cancer survivors (TCS) and controls. The paired Delta-Delta CT method was used for analysis. Each control has a relative value of 1, as they are compared with themselves and each case has a value that is more than one in case of overexpression relative to their paired control, and less than one in case of diminished expression. The column with the value of 1 for controls is included as a visual reference. *p = 0.0479 for the comparison (Wilcoxon signed rank test), mean 1.54 (95% CI of the mean: 1.074–2.005)

The relative expression of CDKN2A*/p16*^*INK4a*^ in total T (CD3+) cells was higher in TCS compared to controls, mean 1.54 (95% CI of the mean: 1.074–2.005), *p* = 0.048. One sample was excluded from this analysis due to extreme values (relative expression > 50). Results are shown in Fig. 1. In order to identify an association between time of NED and CDKN2A*/p16*^*INK4a*^ expression that might represent a temporal overexpression of this molecule, we analyzed the correlation for these two variables, and no association was observed for time of NED and CDKN2A*/p16*^*INK4a*^ expression (r = − 0.37, *p* = 0.174).

## Discussion

The results of this exploratory study showed that testicular cancer survivors (TCS) previously treated with chemotherapy have an immunological phenotype associated with immunosenescence and increased expression of the aging biomarker *p16*^*INK4a*^ in CD3+ lymphocytes. These findings suggest that TCS exposed to chemotherapy might experience premature aging of the immune cells.

Immunological alterations found in TCS after exposure to chemotherapy, revealed a reduced proportion of naïve T cells and a concomitant increment of memory T cells compared to age-matched controls. These changes are similar to those that usually occur in humans as they age and correlate with an immune risk phenotype in the elderly [10].

As humans age, there is an increase in CD57+ terminally differentiated “senescent” cells, which have a reduced proliferative capacity and altered functional properties. TCS, in our study, exhibited an increased percentage in the CD8+ CD45RA+ CD57+ cells (41.6% (22.2–55.6) vs 24.7% (10.1–32.2) *p* = 0.015) compared to controls. TCS exhibited a trend towards an increment in CD28- cells (8.3% vs 1.5%), a group of pro-inflammatory cells that increase gradually with age and may account for up to 50% in the geriatric population. The percentage of CD28-cells in the control group was 1.5% which is within the expected values for the young adults (0.1–2.5%) [8].

In the B lymphocyte subpopulation, there were no significant differences between cases and controls in naïve cells. Interestingly, we documented a lower percentage of memory CD19+ cells in TCS. This phenomenon was not expected as part of the immunosenescence phenotype, besides it could be linked to a reciprocal change in response to primary alterations observed in T cells. Similar changes have been described in patients infected with HIV [16], who present diminished CD4+ counts during the course of the active disease, and perhaps this affects the activation of naïve B cells by cognate T cells, with consequent alteration in the induction and survival of memory B cells [17].

*p16*^*INK4a*^ is a cyclin-dependent kinase inhibitor and an aging biomarker, which renders the senescence growth arrest irreversible. This kinase is encoded by the CDKN2A gene, and it has been shown to be increased in women with breast cancer exposed to chemotherapy. Expression in peripheral blood CD3+ T lymphocytes (PBTLs), has been recognized as a diagnostic marker of senescence in vivo given its very large dynamic range (approximately 16-fold change over a human lifespan), ease and low cost of measure, and strong correlation with chronological age [13, 15, 18–20].

Our study provides insight on the differences of the lymphocyte phenotype and CDKN2A*/p16*^*INK4a*^ expression in a healthy cohort and our TCS population, showing increased expression in the later population. Other factors, such as diabetes and hypogonadism could alter *p16*^*INK4a*^ expression. Type 2 diabetes could potentially bias the results, however, all the participants were tested for fasting glucose, and none of them had diabetes. Additionally, hypogonadism can occur as a side effect in TCS after treatment and could explain an increase of this aging biomarker. Our patients are assessed once a year, and none of the included individuals had criteria for the diagnosis of hypogonadism, and testosterone levels were within normal limits.

This was a cross-sectional study, and we do not know if these alterations remain stable over time. However, TCS were analyzed at different time points after treatment, and there is no evidence of a transitory induction of *p16*^*INK4a*^*.* At inclusion in the study, our TCS had already experienced cancer and oncologic treatment. The causal nature of the effect of chemotherapy versus cancer itself remains to be defined. There were no differences in CDKN2A /*p16*^*INK4a*^ expression between patients that received 3 (*n* = 8) or 4 cycles (n = 8) of BEP (*p* = 0.37) or patients that received one (*n* = 12) or two chemotherapy regimens (*n* = 4) (*p* = 0.93). Future evaluations are planned to be done in a longitudinal study that allows a patient’s evaluation at diagnosis, during and after oncologic treatments. Additionally, this will also allow to discern whether a more intensive chemotherapy regimen (> 3 cycles of BEP, exposure to second-line chemotherapy or high dose chemotherapy) has a greater impact on *p16*^*INK4a*^ expression.

The immunosenescent phenotype is believed to contribute to the development of an immune risk profile in the elderly, associated with an increased rate of infections and a diminished effect of vaccines, and increased cancer susceptibility. The clinical implications of these senescent changes in TCS warrant further investigation. Our findings have added more evidence to potential chemotherapy consequences in the TCS population. We believe this observation reinforces the importance of considering surveillance over chemotherapy or radiation therapy in early stages of the disease. It is possible that premature aging may occur in other organs and tissue compartments of TCS and this is a matter of further study.

## Conclusions

In this exploratory study, TCS exposed to chemotherapy presented multiple alterations in lymphocyte subpopulations and increased expression of CDKN2A /*p16*^*INK4a*^. These alterations are similar to those observed in elderly individuals as part of the immunosenescent phenotype. To our knowledge, there are no previous reports of such a finding in this population. Further studies are warranted in lieu of or findings to define the clinical implications of this premature senescence immunophenotyped in TCS.

## Data Availability

The datasets used and/or analyzed during the current study are available from the corresponding author on reasonable request.

## Abbreviations

TC: Testicular Cancer
IGCCC: International Germ Cell Consensus Classification
TCS: Testicular Cancer Survivors
NK: Natural Killer
NED: No Evidence of Disease
CT: Computed Tomography
BEP: Bleomycin, Etoposide and Cisplatin
PBMC: Peripheral Blood Mononuclear Cells
RT: Reverse Transcriptase
RT-PCR: Real-Time Polymerase Chain Reaction
TCM: Central Memory T Cells
TEM: Effector Memory T Cells
NKT: Natural Killer T Cells
